# Supplementary material for: Capillary Zone Electrophoresis in Tandem with Flow Cytometry in Viability Study of Various ATCC Bacterial Strains under Antibiotic Treatment
Source: Int J Environ Res Public Health. 2022 Feb 6;19(3):1833. doi: 10.3390/ijerph19031833 (PMC8835228; doi:10.3390/ijerph19031833)
Supplement: Supplementary file 1 [file ijerph-19-01833-s001.zip › ijerph-1577263-supplementary.pdf]

***Table S1.*** The value of bacterial cell viability for all tested strains under antibiotic treatment recorded by CZE assay. The data presented in this table have been reported as a results of three repetitions of each with the SD value from 4.62 to 20.05 for *E. faecalis*, from 1.38 to 22.39 for *S. aureus*, from 0.84 to 17.76 for *K. pneumoniae*, from 1.23 to 27.72 for *P. aeruginosa*, and from 0.85 to 23.77 for *E. coli*.

| | STATIC EXPERIMENT | | | | | | | | | | | KINETIC EXPERIMENT | | |
|---|---|---|---|---|---|---|---|---|---|---|---|---|---|---|
| *Enterococcus faecalis ATCC 14506* | | | | | | | | | | | | | | |
| Antibiotic name | Control | Vancomycin | | | | | Cefotaxime | | Metronidazol | Amoxycilin/kl. acid | Clindamycin | Vancomycin, 8 (mg/L) | | |
| concentration (mg/L) | | *0.1* | *1* | *2* | *4* | *8* | *0.5* | *4* | *0.1* | *0.1* | *0.1* | *1h* | *12h* | *24h* |
| % live cells | 95 | 38 | 33 | 33 | 25 | 23 | 75 | 78 | 75 | 65 | 71 | 12 | 2 | 1 |
| *Staphylococcus aureus ATCC 11632* | | | | | | | | | | | | | | |
| Antibiotic name | Control | Metronidazol | | | Clindamycin | Ciprofloxacin | | | | | Amoxicilin | Ciprofloxacin 8 mg/L | | |
| concentration (mg/L) | | *0.1* | *0.5* | *3* | *0.1* | *0.1* | *1* | *2* | *4* | *8* | *0.1* | *1h* | *12h* | *24h* |
| % live cells | 99 | 47 | 85 | 49 | 46 | 37 | 24 | 14 | 3 | 3 | 9 | 17 | 1 | 0 |
| *Klebsiella pneumoniae ATCC 10031* | | | | | | | | | | | | | | |
| Antibiotic name | Control | Vancomycin | | Metronidazol | Amoxycilin/kl. acid | | | | | | Clindamycin | Ciprofloxacin | Amoxycilin/kl. acid 8 (mg/L) | | |
| concentration (mg/L) | | *0.1* | *2* | | *0.1* | *1* | *2* | *4* | *8* | *0.1* | *0.1* | *1h* | *12h* | *24h* |
| % live cells | 95 | 82 | 64 | 75 | 84 | 82 | 75 | 22 | 18 | 87 | 75 | 65 | 1 | 0 |
| *Pseudomonas aeruginosa ATCC 27853* | | | | | | | | | | | | | | |
| Antibiotic name | Control | Cefotaxime | | | | | Metronidazol | Amoxycilin/kl. acid | Clindamycin | Amoxicilin | | Cefotaxime 8 mg/L | | |
| concentration (mg/L) | | *0.5* | *1* | *2* | *4* | *8* | *0.1* | *0.1* | *0.1* | *0.1* | *2* | *1h* | *12h* | *24h* |
| % live cells | 98 | 53 | 40 | 31 | 24 | 17 | 65 | 59 | 79 | 31 | 72 | 73 | 70 | 67 |
| *Escherichia coli ATTC 25922* | | | | | | | | | | | | | | |
| Antibiotic name | Control | Vancomycin | | Cefotaxime | Metronidazol | Amoxycilin/kl. acid | | | | | Amoxicilin | Amoxycilin + kl. acid 8 mg/L | | |
| concentration (mg/L) | | *0.1* | *2* | *0.5* | *0.1* | *0.1* | *1* | *2* | *4* | *8* | *0.1* | *1h* | *12h* | *24h* |
| % live cells | 0 | 0 | 0 | 0 | 0 | 0 | 0 | 0 | 0 | 0 | 0 | 0 | 0 | 0 |

***Table S2.*** The value of bacterial cell viability for all tested strains under antibiotic treatment recorded by FC assay. The data presented in this table have been reported as a results of three repetitions of each with the SD value from 0.09 to 10.47 for *E. faecalis*, from 0.02 to 0.69 for *S. aureus*, from 0.01 to 5.64 for *K. pneumoniae*, from 0.00 to 0.24 for *P. aeruginosa*, and from 0.00 to 0.07 for *E. coli*.

| | STATIC EXPERIMENT | | | | | | | | | | | KINETIC EXPERIMENT | | |
|---|---|---|---|---|---|---|---|---|---|---|---|---|---|---|
| *Enterococcus faecalis ATCC 14506* | | | | | | | | | | | | | | |
| Antibiotic name | Control | Vancomycin | | | | | Cefotaxime | | Metronidazol | Amoxycilin/kl. acid | Clindamycin | Vancomycin, 8 (mg/L) | | |
| concentration (mg/L) | | *0.1* | *1* | *2* | *4* | *8* | *0.5* | *4* | *0.1* | *0.1* | *0.1* | *1h* | *12h* | *24h* |
| % live cells | 96 | 28 | 22 | 20 | 19 | 15 | 74 | 79 | 75 | 67 | 72 | 15 | 16 | 14 |
| *Staphylococcus aureus ATCC 11632* | | | | | | | | | | | | | | |
| Antibiotic name | Control | Metronidazol | | | Clindamycin | Ciprofloxacin | | | | | Amoxicilin | Ciprofloxacin 8 mg/L | | |
| concentration (mg/L) | | *0.1* | *0.5* | *3* | *0.1* | *0.1* | *1* | *2* | *4* | *8* | *0.1* | *1h* | *12h* | *24h* |
| % live cells | 99 | 49 | 79 | 46 | 51 | 46 | 45 | 28 | 0 | 4 | 10 | 35 | 32 | 34 |
| *Klebsiella pneumoniae ATCC 10031* | | | | | | | | | | | | | | |
| Antibiotic name | Control | Vancomycin | | Metronidazol | Amoxycilin/kl. acid | | | | | Clindamycin | Ciprofloxacin | Amoxycilin/kl. acid 8 (mg/L) | | |
| concentration (mg/L) | | *0.1* | *2* | | *0.1* | *1* | *2* | *4* | *8* | *0.1* | *0.1* | *1h* | *12h* | *24h* |
| % live cells | 96 | 81 | 53 | 64 | 84 | 78 | 72 | 27 | 15 | 85 | 63 | 40 | 10 | 8 |
| *Pseudomonas aeruginosa ATCC 27853* | | | | | | | | | | | | | | |
| Antibiotic name | Control | Cefotaxime | | | | | Metronidazol | Amoxycilin/kl. acid | Clindamycin | Amoxicilin | | Cefotaxime 8 mg/L | | |
| concentration (mg/L) | | *0.5* | *1* | *2* | *4* | *8* | *0.1* | *0.1* | *0.1* | *0.1* | *2* | *1h* | *12h* | *24h* |
| % live cells | 99 | 56 | 38 | 29 | 19 | 8 | 64 | 54 | 82 | 30 | 75 | 51 | 50 | 49 |
| *Escherichia coli ATTC 25922* | | | | | | | | | | | | | | |
| Antibiotic name | Control | Vancomycin | | Cefotaxime | Metronidazol | Amoxycilin/kl. acid | | | | | Amoxicilin | Amoxycilin + kl. acid 8 mg/L | | |
| concentration (mg/L) | | *0.1* | *2* | *0.5* | *0.1* | *0.1* | *1* | *2* | *4* | *8* | *0.1* | *1h* | *12h* | *24h* |
| % live cells | 90 | 46 | 38 | 8 | 81 | 68 | 30 | 19 | 14 | 10 | 63 | 40 | 10 | 9 |
